# The effect of cement and impression methods on the marginal and internal adaptation of the current monolithic blocks - 3D scanning evaluation 

**DOI:** 10.4317/jced.61606

**Published:** 2024-07-01

**Authors:** Sebnem Yilbas, Deger Ongul, Burcin Karatasli, Bulent Sermet

**Affiliations:** 1 Graduate student, Istanbul University, Institute of Graduate Studies in Health Sciences Department of Prosthodontics, DDD, PhD, Istanbul, Turkey; 2 Associate Professor Dr. DDS, PhD Department of Prosthodontics, Faculty of Dentistry, Istanbul University; 3 DDS, Ph.D., Department of Prosthodontics, Faculty of Dentistry, Istanbul Nisantasi University; 4 Professor Dr. DDS, PhD Department of Prosthodontics, Faculty of Dentistry, Atlas University

## Abstract

**Background:**

This research aimed to evaluate the marginal and internal gaps of crowns, which were produced using both digital and conventional impression techniques and cemented with various types of cement.

**Material and Methods:**

For the full ceramic crown restoration, an anatomically prepared acrylic first molar phantom tooth (Frasaco GmbH, Germany) was scanned with Scanner S600 ARTI (Zirkonzahn). 160 PMMA analogues produced from the milling unit. Two impression methods were used: digital impressions by intraoral scanner (Aadva Intra Oral 3D Scanner, GC) and PVS impression. Cerasmart, Initial LRF Block, Zirconia Prettau and ICE Zircon monolithic blocks milled with M1 Milling Unit (Zirkonzahn). Restorations cemented with light-cured and dual-cured cements. (n = 10) Pre and post-cementation 3D images overlap was performed using Geomagic Control X (3D Systems, NC, USA). Data were analysed by using SPSS 25.0. *p*<0.05 difference was considered significant.

**Results:**

Digital impressions were significantly higher than PVS impressions in all groups (*p*<0.05). A significant difference was found between the materials (*p*<0.05). Cerasmart showed a significantly more marginal gap than the other groups. Prettau and ICE Zircon crowns with the conventional impression group showed a significantly smaller marginal gap than the others.

**Conclusions:**

Monolithic crowns fabricated by CAD-CAM using the digital and conventional impression methods had clinically acceptable marginal and internal gaps. Crowns cemented with dual-cured cements showed significantly more marginal gap than light-cure groups.

** Key words:**3D scanning, Marginal accuracy, Marginal fit, Monolithic crown.

## Introduction

Dental treatment is moving toward digital technology. One significant innovation has been computer-aided design and computer-aided manufacturing (CAD-CAM), which has gained popularity among dentists over the past 25 years (1). Digital impression (DI) techniques have developed as an alternative to conventional impression methods. Previous research has assessed the accuracy of various intraoral scanners (2). While some studies have shown that these scanners can provide comparable or superior precision to traditional materials like polyvinyl siloxane (PVS) or polyether, others have found that scanners may not be as accurate as conventional impressions (3,4).

The clinically accepTable value for the marginal gap has been discussed in the literature, with proposed values that range from 39 to 120 µm (5). Theoretically, the acceptable marginal discrepancy for cemented crown restorations ranges between 25 and 40 µm; (6-8) however, several *in vitro* studies have reported mean marginal gaps of 64 to 83 µm in CAD/ CAM generated ceramic single-tooth restorations (9-12). The internal gap of ceramic crowns has been reported to be within the range of 123 to 154 µm (13,14).

Zirconia is a material with excellent mechanical properties, which makes it a great alternative to metal frameworks for posterior fixed partial dentures (15-18). All-ceramic crowns are also a good choice for natural-looking teeth, but they can be distorted during fabrication, especially during firing, which can negatively affect their fit (19,20). To address these challenges, polymer-infiltrated ceramic-network (PICN) materials have been developed. These materials are a combination of ceramic, polymer, and zirconia-reinforced lithium silicate ceramic, making them a hybrid ceramic and resin nanoceramic material (21,22).

Different methods are available for measuring marginal gap including silicone replica, stereomicroscopy, scanning electron microscopy, micro-computed tomography and 3D optical scanning (23-27). The purpose of this study was to evaluate a digital means of measuring internal fit and marginal gap with 3D optical scanning and overlapped with a software program (Geomagic Control 2015; 3D Systems). Many reports on DI systems have been published, but only a few have used the GC Intraoral and GC Aadva Lab scanners.

The null hypotheses were as follows: 1) There is no difference in marginal and internal fit between the crowns produced by digital and conventional impressions. 2) There is no difference in marginal and internal fit between different structured ceramics. 3) There is no difference in marginal and internal fit between the crowns cemented with dual-cured and light-cured resin cement.

## Material and Methods

- Preparation of Specimens

**Figure 1:**
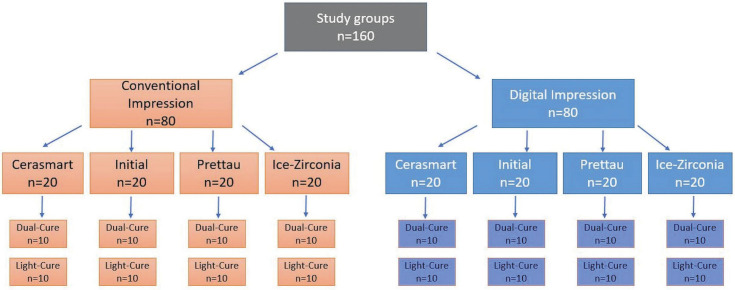
Experimental Groups.

For the preparation of specimens, an anatomically prepared acrylic first molar phantom tooth (Frasaco GmbH, Germany) was used. The acrylic phantom tooth was scanned with Scanner S600 ARTI (Zirkonzahn, Gais / South Tyrol, Italy). From the scanned acrylic tooth model, 160 analogues were prepared from the polymethyl methacrylate (PMMA) based material in the M1 Wet Heavy Metal Milling Unit (Zirkonzahn, Gais / South Tyrol, Italy). Groups are named as in Figure 1.

-Conventional and Digital Impression Methods 

To compare the effect of conventional impression (CI) and digital impression (DI) methods on marginal adaptation, the specimens were divided into two subgroups. All impressions was taken by the same clinician. For the CI group, the acrylic teeth were embedded in acrylic to a 2 mm apical portion of the step border in a die-casting mold one by one. (Paladur, Kulzer, Wehrheim, Germany). The impressions of the acrylic teeth were taken with polyvinyl siloxane impression material (Betasil Vario Putty / Light, Müller- Omicron, Germany). Type IV dental gypsum (Silky-Rock, Whip- Mix Corporation, Louisville, USA) was poured to obtain the casts. Gypsum casts were scanned with AAdva Lab Scan (GC Europe NV, Leuven) and recorded in STL format. The whole workflow was performed by the same laboratory. In the second group, 80 acrylic teeth impressions were recorded with DI method with GC Aadva Intra Oral 3D Scanner (GC Europe NV. Leuven). All data were recorded in STL format and sent to the dental laboratory. STL files from both groups were used to design crowns using the software (Zirkonzahn Archiv, Zirkonzahn, Steger, Ahrntal, Italy).

-Production of Crown 

Four different monolithic block groups were used in this study as listed in Figure 2. Design of 3D anatomical substructure of the digital restorations was produced by CAD/CAM Software System (Zirkonzahn Archiv, Zirkonzahn, Steger, Italy) for all specimens. All crown restorations were milled at the M1 Wet Heavy Metal Milling Unit (Zirkonzahn, Gais / South Tyrol, Italy).

-Cementation of Specimens

To compare the effect of light-cured(LC) and dual-cured(DC) cements on marginal adaptation, restorations in all groups are divided into two subgroups: light-cured G-aenial Universal Flow (GC, Leuven, Belgium) and dual-cured G-CEM Link Force (GC, Leuven, Belgium) cements. (n = 10) 

For the (DC) cement group, DC cement set (G-Cem Link Force, Dual-cure Adhesive Luting Cement, GC, Leuven, Belgium) was used. The cementation steps of the specimens were carried out according to the manufacturer’s instructions. The inner surface of the crown prosthesis etched with hydrofluoric acid, G- Multi Primer (GC, Leuven, Belgium) silane was applied, G- Premio Bond (GC, Leuven, Belgium) and DCA (GC, Leuven, Belgium) were mixed and applied on the analogue. The G-CEM Link Force (GC, Leuven, Belgium) was applied to the restoration with a cement syringe.

For the (LC) cement group, inner surface of the crown prosthesis etched with hydrofluoric acid, G-Multi Primer (GC, Leuven, Belgium) silane was applied, G-Premio Bond (GC, Leuven, Belgium) was applied to the analogue. The LED light device (EliparTM S10, 3M ESPE GmbH, Seefeld, Germany) was applied for 10 seconds. The light-cured G- aenial Universal Flow (GC, Leuven, Belgium) was applied to the restoration with a cement syringe. After cementation; a static force of 50 N was applied to the specimens by a metal weighting device to apply a constant force to the specimens during the cementation step. LED light device at a wavelength of 430 - 480 nm was applied to finish the cementation (EliparTM S10, 3M ESPE GmbH, Seefeld, Germany).

-Marginal and Internal Fit Measurement

Pre-cementation and post-cementation images of the crown restorations and analogues were scanned by the Solutionix C500 3D Scanner (Solutionix, Seoul, Korea). The Solutionix C500 consists of 2, 5 megapixels high-resolution cameras that maximize the quality of 3D scanning data. The 3D image overlap was performed using Geomagic Control X (3D Systems, NC, USA) and Autodesk Powershape software. The marginal gap (µm) and internal fit (mm3) were evaluated.

-Statistical analysis

Data were analyzed by using SPSS 25.0 (Statistical Packages of Social Sciences). The data for normal distribution was evaluated by Kolmogorov Smirnov test. Descriptive statistics were shown as mean ± standard deviation, median, minimum and maximum value for continuous variables. Mann Whitney U test was used to compare the variables that did not fit the normal distribution of the two independent groups. Kruskal-Wallis test was used for comparison of variables that did not fit the normal distribution of more than two groups, and Mann-Whitney U test which was post-hoc test was used for pairwise comparison of statistically significant variables. If *p* <0.05, the difference was considered significant.

## Results

Regardless of the material and cement type used, the marginal and internal gap of the (CI) method is smaller than the (DI) method, ( Table 1, Table 2). A statistically significant difference was found between digital and conventional impression methods (*p* <0.05). In the (DI) group, the marginal gap and internal fit of the (DC) cement group were statistically significant compared to the (LC) cement group (*p* <0.05) ( Table 3, Table 4). The marginal gap of the (LC) cement group (102 μm) is smaller than the (DC) cement group (121 μm). The internal fit of the (LC) cement group (24 mm³) was superior to the (DC) cement group (29 mm³).

In the (DI) group, there was a statistically significant difference between the materials (*p* <0.05). A significant difference was found between Prettau-Cerasmart (*p* = 0.016) and Prettau-Zircon (*p* = 0.006) materials. The lowest marginal gap value was found in Prettau material (98 μm). In the (CI) group, there is a statistically significant difference between the materials (*p* <0.05). Initial-Zircon (*p* = 0,000), Cerasmart-Zircon (*p* = 0,000), Prettau-Cerasmart (*p* = 0,000) and Prettau-Initial (*p* = 0.019) materials were found to be significantly different. The smallest marginal gap value was found in the Prettau material (64 μm). The highest marginal gap value was found in Cerasmart (108 μm) material ( Table 5).

## Discussion

The aim of this *in vitro* study was to evaluate the marginal fit of four monolithic crowns produced from two impression techniques and cemented using two different kinds of resin cement. Based on the results, the null hypothesis that the impression methods (conventional and digital), monolithic crown groups and cement types (dual-cure and light-cure) would not affect the marginal and internal fit was rejected.

In this study, the DI; (111 μm) showed significantly more marginal gap than the CI groups (78 μm). Therefore, the null hypothesis regarding the difference in the clinical marginal fit of crowns was rejected. These result supports the previous study which evaluate the marginal fit of all-ceramic crowns produced from two impression techniques (digital vs. conventional) with using Geomagic Studio 2012 software. In the study, the DI group showed a statistically significantly less accurate marginal adaptation than the CI groups (28). The study found that while there was a statistical difference in the accuracy of crowns made from traditional PVS impressions versus digital impressions, this difference may not have significant clinical implications. This is because all of the marginal gaps observed in both methods were less than the clinically acceptable limit of 120 μm.

In a meta-analysis study which evaluate the marginal fit of crown restorations produced after DI and CI methods; in-vitro studies showed that the mean value of CI fit value was 58.9 μm, whereas the mean value of DI fit value was 63.3 μm (29). In the current study, hybrid ceramics - Cerasmart, leucite reinforced glass ceramics - Initial LRF Block, fully anatomically processed zirconia - Zirconia Prettau and layered zirconia - ICE Zircon blocks were used. There is a statistically difference between block groups, Therefore, the null hypothesis that there is no difference between the clinical marginal fit of crowns in four different block groups was rejected. The marginal range of Cerasmart material is statistically higher than that of Initial block. The micro-hardness of Cerasmart composite resin materials is softer than that of Initial glass ceramic materials, which affects milling precision and marginal compatibility. In the study which investigate the mechanical properties of composite resin materials and glass ceramic materials, micro-hardness test results showed that composite resin materials (Cerasmart, Lava Ultimate) were softer than glass ceramic materials. The hardness of the materials contributes to the ease of milling. The results of our study support the results of this study (30).

The chemical structure of restorative CAD-CAM material has a significant impact on the fit of the restoration, both marginally and internally. Previous research has indicated that materials with low stiffness and modulus of elasticity result in the removal of more material during milling (30). On the other hand, some studies have suggested that less brittle materials are less prone to chipping, easier to machine, and provide better adaptability (31,32). In our study, when four different materials were compared with each other regardless of the impression methods and cement types, a significant difference was found between the materials (*p* <0.05). According to this; Prettau-Cerasmart (81 μm – 113 μm), Zircon-Cerasmart (89 μm – 113 μm) and Initial-Cerasmart (96 μm - 113 μm) materials were found to be significantly different. The marginal gap of Cerasmart material is higher than other materials. An interesting finding in this study was that the Prettau crowns from (DI) had significantly smaller marginal gap than the other crowns. Prettau and Zircon crowns from (CI) group had significantly smaller marginal gap than the other crowns. The reason for the difference is because the other studies mainly used CEREC Inlab and different milling devices, while Zirkonzahn Milling Machine was used in the current study. All specimens were produced using Zirkonzahn Milling Machine. Hybrid ceramics aim to combine the high durability advantage of ceramic with the flexibility and aesthetic advantages of the composite As there aren’t enough researchers for these newly developed materials, our study aims to support insufficient studies and close the gap in this area.

There is a statistically significant difference between the different cement groups. Therefore, the last null hypothesis, which states that there is no difference between two different resin cement groups in the clinical marginal fit of crowns, was rejected. In the (DI) group, the marginal gap and internal fit of the (DC) cement group was statistically significant compared to the LC cement group (*p* <0.05). The marginal gap of the LC cement group (102 μm) is smaller than the DC cement group (121 μm). The internal gap of the LC cement group (24 mm³) was smaller than the DC cement group (29 mm³). In a previous study, different resin cements were evaluated for their marginal gap in restorations after cementation. The resin cement thickness in DC were found as 65 μm and 82 μm in LC cement group. This study suggests that the light curing source may have difficulty reaching deep proximal areas, which could account for the larger marginal gap in the dual-cure cement group compared to the light-cure cement group (33). In a previous study, the marginal gap of crowns was evaluated after scanning with the CEREC Omnicam intraoral scanner and Cerec in-lab scan, and then production by the CEREC MC XL milling unit (34). Crowns were cemented using self-adhesive resin cement by finger pressure (34). The marginal gap measurement for the intraoral scanner group was 115 μm, while the lab scan group had a measurement of 130 μm. In the current study, both DI and CI groups showed smaller gap values as a result of using a 50 N (≈ 5 kg) static force to apply constant force to the specimens during the cementation phase for standardization purposes.

The limitation of the study is that thermal cycle was not applied to the groups, which may affect the results of clinical usage. Further *in-vivo* and *in-vitro* research is necessary to facilitate the use of newly developed ceramic materials.

## Conclusions

Within the limitations of this *in vitro* study, the following conclusions were drawn.

1. The marginal and internal gap values of the digital impression method were higher than the conventional impression method.

2. The marginal and internal gap values of the dual-cure cement group were higher than the light-cure cement group.

3. The combination of zirconia based material, conventional impression, and light-cured cementation produced the most accurate marginal fit for all-ceramic restorations.

## Figures and Tables

**Table 1:** Marginal gap of digital and conventional impression methods (in µm).

Group	N	Mean	Std.Dev.	Median	Min.	Max.	P
Digital	80	,111	,022	,111	,061	,168	0,000*
Conventional	80	,078	,025	,072	,042	,130	
Total	160	,095	,029	,093	,042	,168	-

Mann-Whitney U test * *p*<0.05 is statistically significant.

**Table 2:** Internal gap of digital and conventional impression methods (in mm³).

Group	N	Mean	Std.Dev.	Median	Min.	Max.	P
Digital	80	26,82	5,66	27,02	10,00	39,92	0,000*
Conventional	80	21,73	6,23	20,74	10,57	39,83	
Total	160	24,28	6,46	23,24	10,00	39,92	-

Mann-Whitney U test * *p*<0.05 is statistically significant.

**Table 3:** Marginal gap of dual-cure and light-cure cements in groups of digital and conventional impression methods (in µm).

Group	Cement	N	Mean	Std.Dev.	Median	Min.	Max.	P
Digital	Dual	40	,121	,019	,121	,084	,168	0,000*
	Light	40	,102	,022	,093	,061	,155	
	Total	80	,111	,022	,111	,061	,168	-
Conventional	Dual	40	,078	,025	,068	,042	,119	1,000
	Light	40	,078	,025	,074	,042	,130	
	Total	80	,078	,025	,072	,042	,130	-

Mann-Whitney U test * *p*<0.05 is statistically significant.

**Table 4:** Internal gap of dual-cured and light-cured cements in groups of digital and conventional impression methods (in mm³).

Group	Cement	N	Mean	Std.Dev.	Median	Min.	Max.	P
Digital	Dual	40	29,58	5,30	29,00	19,69	39,92	0,000*
	Light	40	24,05	4,59	23,79	10,00	35,38	
	Total	80	26,82	5,66	27,02	10,00	39,92
Conventional	Dual	40	21,28	6,75	20,51	13,02	39,83	0,312
	Light	40	22,19	5,71	21,96	10,57	34,89	
	Total	80	21,73	6,23	20,74	10,57	39,83

Mann-Whitney U test * *p*<0.05 is statistically significant.

**Table 5:** Marginal gap of materials in groups of digital and conventional impression methods (in µm).

Group	Material	N	Mean	Std.Dev.	Median	Min.	Max.	P
Digital	Prettau	20	,098	,019	,091	,062	,148	0,003*
	Cerasmart	20	,119	,022	,131	,084	,155	
	Initial	20	,106	,016	,106	,061	,131	
	ICE- Zir	20	,122	,024	,122	,086	,168	
	Total	80	,111	,022	,111	,061	,168
Conventional	Prettau	20	,064	,017	,056	,045	,104	0,000*
	Cerasmart	20	,108	,015	,110	,072	,130	
	Initial	20	,086	,016	,084	,064	,112	
	Ice-Zir	20	,056	,011	,054	,042	,091	
	Total	80	,078	,025	,072	,042	,130

Kruskal Wallis test * *p*<0.05 is statistically significant.

## Data Availability

The datasets used and/or analyzed during the current study are available from the corresponding author.
